# Updating trends in sweetened beverages consumption in Brazil from 2007 to 2021

**DOI:** 10.11606/s1518-8787.2024058005661

**Published:** 2024-09-09

**Authors:** Luiza Eunice Sá da Silva, Thaís Cristina Marquezine Caldeira, Taciana Maia Sousa, Rafael Moreira Claro

**Affiliations:** I Universidade Federal de Minas Gerais. Faculdade de Medicina. Belo Horizonte, MG, Brasil; II Universidade do Estado do Rio de Janeiro. Instituto de Nutrição. Departamento de Nutrição Social. Rio de Janeiro, RJ, Brasil; III Universidade Federal de Minas Gerais. Escola de Enfermagem. Departamento de Nutrição. Belo Horizonte, MG, Brasil

**Keywords:** Sweetened Beverages, Health Surveys, Chronic Diseases, Public Health

## Abstract

**OBJECTIVE:**

To analyze the time trend of sweetened beverages consumption among Brazilian adults in 26 capitals and the Federal District, from 2007 to 2021, with focus on the most recent period (2015 to 2021).

**METHODS:**

Data from the *Sistema de Vigilância de Fatores de Risco e Proteção para Doenças Crônicas por Inquérito Telefônico* (Vigitel - Surveillance System of Risk and Protection Factors for Chronic Diseases by Telephone Survey)were used to conduct a time-series analysis (n = 731,683). The prevalence of regular consumption (five or more days/week), average daily consumption (milliliters) and nonconsumption of sweetened beverages were analyzed. Prais-Winsten regression models were used to calculate temporal trends of the indicators for the complete set of the evaluated population and by sociodemographic characteristics (sex, age group, schooling and development level of the geographic region of residence).

**RESULTS:**

Between 2007 and 2021, a reduction in the prevalence of regular consumption (-1.23 pp/year) and daily average consumption (-8.62 milliliters/year) of sweetened beverages was observed. However, between 2015 and 2021, this downward trend did not continue. The prevalence of adults who reported not consuming sweetened beverages increased (1.14 pp/year, for 2007–21), although this trend was not significant in the most recent period.

**CONCLUSIONS:**

The consumption of sweetened beverages among Brazilian adults decreased in the 15 years studied. However, this reduction was not observed more recently, suggesting that further actions must be adopted in the country so that the trend observed in the total period is maintained.

## INTRODUCTION

Sweetened beverages (SB) have been widely associated with excessive weight gain and non-communicable diseases (NCD)^1^. SB consumption is related to unhealthy diets, considering that sugar is consumed usually in large quantities, increasing the diet’s energy density. In addition, the calories provided by SB have lower nutritional value and provide less satiety than solid foods^1,2^. The consumption of artificially SB, such as diet, light and zero drinks, also indicates potential health risks^3^. Therefore, public policies to reduce the consumption of SB have been proposed and enforced by the World Health Organization (WHO) for several years^4^. The examples include SB taxation, restrictions on SB marketing and promotion of nutritional education actions to decrease SB consumption as well as combat obesity and NCD, which are already in force in some countries^4,5^.

SB consumption varies by gender, age, geographic location and socio-economic status, with a higher prevalence among men, young adults, people with lower education level and residing in metropolitan areas^6,7^. Brazil experienced a significant reduction in SB consumption between 2007 and 2016^8^. While the causes of such reduction are not entirely clear, this may be attributed to increased awareness of the health risks involved in the SB consumption, especially due to an intensification of actions in primary health care^9-11^ and the publication of clear dietary guidelines^12^. On the other hand, it was noted that the prevalence of Brazilians who consumed these drinks on five or more days of the week remained high (16.5%)^8^. Furthermore, most initiatives have been weakened or even stopped during the political and economic crisis experienced by the country since 2015, potentially impacting the reduction in SB consumption observed until that moment. There has been a reduction in investments in social and health policies, impacting several health indicators, along with an increase in unemployment and inflation^13,14^.

In addition, the continuous surveillance of consumption is also justified by the recent ongoing discussion on tax reform in Brazil^15^. What is expected in this new reform is to make the system fairer and guarantee selective taxes on products harmful to health, penalizing sectors that profit at the expense of the health of the population and the environment^15^, as already indicated by international health entities^16^ and already applied in several countries with positive results in this context. Thus, our study aimed to analyze a time trend in SB consumption among Brazilian adults in the 26 capitals and Federal District, from 2007 to 2021, with a special focus on the most recent period (2015 to 2021).

## METHODS

### Study Population and Sampling

Data from the *Sistema de Vigilância de Fatores de Risco e Proteção para Doenças Crônicas por Inquérito Telefônico* (Vigitel - Surveillance System of Risk and Protection Factors for Chronic Diseases by Telephone Survey), between the years of 2007 and 2021 (n = 731,683), were used to conduct a time-series analysis. This system was implemented in 2006 by the Brazilian Ministry of Health (MoH) to collect self-reported health information of risk and protective factors for NCD among the adult population (≥ 18 years) of Brazilian capitals and Federal District^17^.

From 2007 to 2019, the system established a minimum sample size of approximately 2 thousand interviews annually in each city to obtain, with a 95% confidence interval and a maximum error of 2%, the frequency of any factor in the adult population. In 2020 and 2021, due to the difficulties imposed by the COVID-19 pandemic to data collection, a minimum sample size of one thousand individuals was used, increasing the expected sampling error to 4%. A probabilistic sample of adults living in households served by at least one landline telephone is drawn in each city per year. The sampling process consisted of a random selection of 10 thousand landline telephones per city, with the identification of the eligible landline performed in up to six attempts to contact on distinct days and hours. Then, one individual was randomly selected for the interview, from among all adults in the household^17^. More information on the sampling process and data collection applied by Vigitel can be found in the annual reports^17^.

### Data Collection and Organization

The questionnaire was structured to allow a computer-assisted telephone interviewing system. SB consumption was investigated based on the following questions:

- "How many days a week do you usually drink soft drinks or artificial juice? (1–2; 3–4; 5–6 days/week; every day, including weekends; almost never; never)" for regular consumption (≥ 5 days/week, irrespective of the quantity consumed); and
- "How many glasses/cans do you usually drink per day? (1; 2; 3; 4; 5; 6 or more; I don’t know)" for average daily SB consumption (milliliters *per capita*).

The latter question was not collected by Vigitel in 2017, therefore it was not possible to analyze that year. The average daily consumption was calculated by multiplying the number of days of the week that the consumption was reported (mean value of the reported range) by the average amount consumed on a day (considering a glass equal to 300 milliliters), and then dividing the result by the number of days in a week. This indicator considered only individuals with ≥ 1 days of SB consumption per week. Nonconsumption of SB (consumption reported as "almost never" and "never") was also considered.

The analyses were complemented with sociodemographic variables: sex (male; female), age group (18–24; 25–34; 35–44; 45–54; 55–64; ≥ 65 years), schooling (0–8; 9–11; ≥ 12 years), and development level of the geographic region of residence (less developed regions: North and Northeast; more developed regions: Midwest, Southeast and South).

### Data Analysis

The prevalence of regular SB consumption, the daily average consumption of SB (milliliters *per capita*) and the prevalence of nonconsumption of SB were then estimated for each year. Prais-Winsten regression models were conducted to investigate the presence of significant linear trends for the total population and according to sociodemographic strata. The statistical significance of the indicator’s trend in the period was assessed through the regression coefficient, indicating the average annual rate of increase or decrease for each indicator, expressed in percentage points per year (pp/year) for the prevalence of regular or nonconsumption of SB, and milliliters per year (mL/year) for the daily average consumption of SB. We considered significant those variations corresponding to a regression coefficient statistically different from zero (p < 0.05).

Vigitel data includes weighting factors, one to correct the unequal probability of selection (when the household has more than one landline telephone or more than one resident), and another to equate the distribution of the population interviewed in each city (by sex, age and education) to its entire population (based on census data and official projections for the population)^17^.

Data organization, processing and statistical analyses were conducted with Stata software, version 16.1, considering the design of the Vigitel sample.

Vigitel data is available for public access and use from the official page of the MoH and do not allow the identification of interviewees (https://svs.aids.gov.br/download/Vigitel). The data collection was authorized by the Brazilian Ethics Committee of the MoH (number 65610017.1.0000.0008) in June 2017.

## RESULTS

A total of 731,683 Brazilian adults were interviewed from 2007 to 2021. A decrease on regular consumption of SB, from 30.9% to 14.0% (-1.23 pp/year [p < 0.05]) was observed for the total population. Such decrease was greater among men (-1.34 pp/year [p < 0.05]), adults aged between 18 and 44 years (18–24 years: -1.60 pp/year [p < 0.05]; 25–34 years: -1.46 pp/year [p < 0.05]; 35–44 years: -1.21 %/year [p < 0.05]), with more than nine years of study (9–11 years: -1.35 pp/year [p < 0.05]; 12 or more years: -1.36 pp/year [p < 0.05]) and residents of less developed regions (-1.30 pp/year [p < 0.05]). Considering the most recent period (2015–2021), reduction was observed only among two age groups (18–24 and 35–44 years), those with 9–11 years of schooling and those from less developed regions, with a lower magnitude of reduction (Table 1).

**Table 1:** Prevalence of regular consumption (≥ 5 days/week) of sweetened beverages among Brazilian adults (aged ≥ 18 years) by sociodemographic variables. Vigitel, 2007–2021.

Variable		Prevalence of the regular SB consumption (%)															Coef. 2007–2021^a^	Coef. 2015–2021^b^
		2007	2008	2009	2010	2011	2012	2013	2014	2015	2016	2017	2018	2019	2020	2021		
Sex																		
	Male	35.7	30.7	29.3	30.0	32.0	29.8	26.7	23.9	22.4	19.6	17.4	17.7	18.3	17.9	17.2	-1.34^c^	-0.75
	Female	26.9	22.8	23.2	24.1	23.6	22.7	20.4	18.2	16.1	13.9	12.2	11.6	12.3	12.8	11.3	-1.15^c^	-0.69
Age group (years)																		
	18 to 24	43.2	36.3	36.8	35.3	39.6	36.3	33.2	28.9	30.2	24.2	22.8	23.4	22.4	22.1	19.7	-1.60^c^	-1.16^c^
	25 to 34	37.3	34.3	32.2	34.1	32.8	31.9	29.8	25.9	23.8	20.1	17.0	18.1	19.3	17.6	18.3	-1.46^c^	-0.74
	35 to 44	29.9	25.2	25.3	29.4	26.4	26.6	24.1	21.7	17.9	16.9	15.2	14.9	15.8	15.4	13.1	-1.21^c^	-0.60^c^
	45 to 54	24.6	20.3	20.0	20.4	22.8	21.6	17.5	17.8	14.2	12.7	12.4	10.9	10.9	13.6	11.9	-0.94^c^	-0.24
	55 to 64	18.3	16.2	16.0	14.3	18.6	15.8	13.2	11.8	11.9	10.5	8.8	7.8	9.6	9.9	9.1	-0.71^c^	-0.37
	≥ 65	17.0	11.2	13.0	12.9	14.1	12.1	11.4	10.1	9.4	9.9	7.8	7.0	8.4	10.0	8.7	-0.49^c^	-0.05
Schooling (years)																		
	0 to 8	28.1	23.6	22.8	22.9	25.3	24.9	22.1	18.0	16.6	15.9	13.3	12.2	13.5	15.3	12.6	-1.10^c^	-0.52
	9 to 11	34.4	29.6	29.0	30.7	30.3	27.9	25.8	24.4	22.5	18.7	17.0	17.0	17.3	16.4	16.1	-1.35^c^	-0.93^c^
	≥ 12	31.1	27.0	27.1	27.6	26.6	24.5	21.1	19.4	16.9	14.6	13.2	13.4	13.8	13.7	12.7	-1.36^c^	-0.59
Geographic region																		
	North, Northeast	27.1	20.1	20.7	21.1	21.2	19.9	18.6	15.8	14.5	11.2	9.9	8.6	8.7	7.8	9.0	-1.30^c^	-0.88^c^
	Midwest, Southeast and South	32.9	29.8	28.8	29.9	30.9	29.2	25.8	23.5	21.4	19.4	17.2	17.6	18.5	19.3	16.8	-1.19^c^	-0.54
Total		30.9	26.4	26.0	26.8	27.5	26.0	23.3	20.8	19.0	16.5	14.6	14.4	15.0	15.2	14.0	-1.23^c^	-0.73

Vigitel: Surveillance System of Risk and Protective Factors for Chronic Diseases by Telephone Survey; SB: sweetened beverages.

Note: Weighted percentage to adjust the sociodemographic distribution of the Vigitel sample to the distribution of the adult population of each city estimated for each year of study.

a Corresponding to the Prais-Winsten regression coefficient value of the indicator in 2007–2021 (expressed in percentage points per year).

b Corresponding to the Prais-Winsten regression coefficient value of the indicator in 2015–2021 (expressed in percentage points per year).

c p < 0.05.

A similar scenario was observed regarding the daily average consumption (milliliters *per capita*) of SB. At the beginning of the period (2007), Brazilian adults consumed a mean 430.4 mL of SB per day and, in 2021, this consumption reduced to 287.4 mL (-8.62 mL/year [p < 0.05]). The decline was more intense among men (-10.32 mL/year [p < 0.05]), those aged between 18 to 34 years (18–24 years: -10.48 mL/year [p < 0.05]; 25–34 years: -9.74 mL/year [p < 0.05]) and those with 9–11 years of schooling (-9.09 mL/year [p < 0.05]). A significant reduction for the recent period (2015–2021) was observed only among men, individuals aged 18–24 years and those from less developed regions, with a lower magnitude of reduction (Table 2). Finally, the nonconsumption of SB increased for the total population (from 30.4% in 2007 to 41.3% in 2021, 1.14 pp/year [p < 0.05], in all strata (except for individuals aged ≥ 65 years). This increase was greater in women (1.23 pp/year [p < 0.05]), those aged 18–44 years (18–24 years: 1.12 pp/year [p < 0.05]; 25–34 years: 1.12 pp/year [p < 0.05]; 35–44 years: 1.12 pp/year [p < 0.05]), with ≥ 12 years of study (1.52 pp/year [p < 0.05]) and residents of less developed regions (1.38 pp/year [p < 0.05]). Similar to the indicators presented above, the increase in the most recent period was significant only among men (1.10 pp/year [p < 0.05]) (Table 3).

**Table 2:** Daily average consumption of sweetened beverages among Brazilian adults (aged ≥ 18 years) by sociodemographic variables. Vigitel, 2007–2021.

Variable		Average daily consumption of SB (milliliters)															Coef. 2007–2021^a^	Coef. 2015–2021^b^
		2007	2008	2009	2010	2011	2012	2013	2014	2015	2016	2017	2018	2019	2020	2021		
Sex																		
	Male	500.3	418.7	418.8	408.0	424.9	407.1	389.3	351.4	362.2	327.4	–	333.0	329.2	333.9	326.5	-10.32^c^	-3.04^c^
	Female	362.7	318.5	317.9	324.8	319.8	316.7	294.8	294.2	275.1	247.1	–	252.1	261.1	274.1	247.6	-7.16^c^	0.43
Age group (years)																		
	18 to 24	509.9	446.0	448.4	419.6	462.9	445.4	405.7	382.2	394.7	350.6	–	368.2	358.9	352.1	327.7	-10.48^c^	-8.25^c^
	25 to 34	465.3	414.3	400.5	401.3	408.5	396.4	382.5	364.9	351.3	311.3	–	315.4	325.1	327.2	327.6	-9.74^c^	-0.93
	35 to 44	407.8	345.1	344.9	375.4	347.0	368.8	341.0	310.5	306.6	282.7	–	290.3	314.7	308.0	282.9	-6.91^c^	1.31
	45 to 54	370.7	299.9	318.3	314.2	320.1	293.9	283.4	280.7	268.0	256.4	–	262.1	248.3	287.0	257.9	-5.82^c^	1.67
	55 to 64	327.5	269.1	287.7	274.9	283.3	278.8	250.9	232.2	242.0	227.2	–	214.6	226.2	239.3	226.8	-6.02^c^	-0.72
	≥ 65	308.6	240.9	245.0	243.5	248.0	218.1	230.3	222.2	213.0	207.7	–	196.6	195.9	220.0	209.4	-4.92^c^	0.09
Schooling (years)																		
	0 to 8	432.5	357.8	357.4	358.9	376.0	363.7	350.5	307.0	314.9	295.9	–	292.5	289.0	325.4	301.8	-7.55^c^	0.80
	9 to 11	450.8	397.7	387.0	396.3	398.9	377.0	366.6	357.7	347.9	301.1	–	318.1	325.5	316.5	300.5	-9.09^c^	-3.05
	≥ 12	390.3	336.4	350.9	328.2	320.9	331.6	294.7	287.8	281.6	262.9	–	264.4	265.7	271.2	260.9	-8.11^c^	-1.16
Geographic region																		
	North, Northeast	398.9	320.0	330.9	326.8	324.6	314.7	307.3	289.2	289.2	250.7	–	247.7	244.0	235.0	258.0	-8.95^c^	-5.52^c^
	Midwest, Southeast and South	446.1	392.8	386.2	385.6	395.3	385.2	360.3	340.3	334.9	306.3	–	316.6	320.7	335.6	301.9	-8.86^c^	0.11
Total		430.4	367.9	367.2	365.4	370.8	361.0	341.9	322.9	319.4	287.6	–	294.2	296.2	303.8	287.4	-8.62^c^	-1.37

Vigitel: Surveillance System of Risk and Protective Factors for Chronic Diseases by Telephone Survey; SB: sweetened beverages.

Note: information on the number of glasses/cans usually consumed was not collected in 2017.

a Corresponding to the Prais-Winsten regression coefficient value of the indicator in 2007–2021 [expressed in percentage points per year (pp/year)].

b Corresponding to the Prais-Winsten regression coefficient value of the indicator in 2015–2021 (expressed in percentage points per year).

c p < 0.05.

**Table 3:** Prevalence of nonconsumption of sweetened beverages among the Brazilian adults (aged ≥ 18 years) by sociodemographic variables. Vigitel, 2007–2021.

Variable		Prevalence of nonconsumption of sweetened beverages (%)															Coef. 2007–2021^a^	Coef. 2015–2021^b^
		2007	2008	2009	2010	2011	2012	2013	2014	2015	2016	2017	2018	2019	2020	2021		
Sex																		
	Male	25.9	21.9	20.9	19.1	20.0	21.2	25.1	27.8	31.1	28.0	32.2	36.5	33.5	35.1	35.6	1.05^c^	1.10^c^
	Female	34.3	31.2	29.1	27.5	27.5	29.9	35.5	38.6	43.0	39.5	45.6	50.2	46.8	43.9	46.3	1.23^c^	0.68
Age group (years)																		
	18 to 24	13.5	12.4	10.9	10.7	10.4	12.9	15.8	18.8	20.2	20.7	25.0	26.0	25.7	22.5	26.6	1.12^c^	0.85
	25 to 34	20.5	17.0	16.2	13.2	15.0	15.8	19.7	23.6	26.9	26.0	30.9	34.3	31.2	30.7	32.3	1.12^c^	0.93
	35 to 44	29.3	23.3	22.0	20.2	21.3	23.9	27.0	28.9	33.8	30.5	35.6	40.0	39.0	35.9	38.5	1.12^c^	1.00
	45 to 54	38.3	34.7	32.8	28.9	29.0	30.9	37.6	38.6	44.5	39.8	44.7	51.0	46.1	47.5	45.4	1.02^c^	0.77
	55 to 64	50.3	43.0	41.8	41.3	37.3	41.5	47.6	50.3	54.4	48.3	54.3	60.1	53.9	53.5	53.9	0.88^c^	0.33
	≥ 65	56.5	56.3	51.0	50.9	51.7	49.8	57.4	61.5	63.4	53.3	58.7	64.6	59.1	58.4	61.2	0.60	0.32
Schooling (years)																		
	0 to 8	38.6	33.9	31.6	30.4	31.2	31.9	37.9	41.4	45.1	38.6	44.7	50.8	47.2	45.1	48.3	1.16^c^	0.94
	9 to 11	23.7	20.9	20.3	19.3	18.8	21.4	25.8	27.8	31.6	29.5	36.0	38.5	36.7	35.4	37.2	1.28^c^	1.03
	≥ 12	23.9	22.3	21.6	18.6	20.4	24.1	27.8	31.6	36.2	35.0	38.2	43.7	39.7	40.8	40.7	1.52^c^	0.95
Geographic region																		
	North, Northeast	31.2	28.0	26.5	24.6	24.8	27.4	31.4	35.7	39.7	37.3	43.7	48.6	46.8	46.7	46.0	1.38^c^	1.38
	Midwest, Southeast and South	30.0	26.3	24.7	23.1	23.6	25.1	30.4	32.5	36.3	32.6	37.1	41.3	37.3	36.1	38.8	0.98^c^	0.53
Total		30.4	26.9	25.3	23.6	24.0	25.9	30.7	33.7	37.5	34.2	39.4	43.9	40.7	39.8	41.3	1.14^c^	0.85

Vigitel: Surveillance System of Risk and Protective Factors for Chronic Diseases by Telephone Survey.

Note: Weighted percentage to adjust the sociodemographic distribution of the Vigitel sample to the distribution of the adult population of each city estimated for each year of study.

a Corresponding to the Prais-Winsten regression coefficient value of the indicator in 2007–2021 (expressed in percentage points per year).

b Corresponding to the Prais-Winsten regression coefficient value of the indicator in 2015–2021 (expressed in percentage points per year).

c p < 0.05.

## DISCUSSION

Trend analyses of SB consumption in Brazil in the past 15 years, with more than 730 thousand interviews, exposed relevant results. Initially, they confirmed the reduction in SB consumption for the entire period (2007–2021). This reduction was consistent (observed in two different indicators—percentage of regular SB consumption and daily average SB consumption), and in all population strata, mainly until 2014. An analysis of the most recent period (2015–2021) showed the stagnation of these indicators, allowing us to conclude that the reduction is essentially due to the period between 2007 and 2014.

Although the recent period (2015–2021) includes only seven years, demanding caution in the analysis of observed trends, the stagnation of indicators in this period should be considered with a view to strengthen public health actions in the country. It is worth mentioning that this evidence must be seen in a context in which other studies analyzing the trend of other risk factors already identified a stagnation or even reversion of positive trends^14,18,19^.

A previous study from our research group examined data from the same population and reported a decrease in SB consumption between 2007 and 2016, and the downward trend remained significant in the most recent period (2012–2016)^8^. By that moment, this positive result was associated with the intensification of actions at the level of primary health care in Brazil, such as the inclusion of dietitians in multidisciplinary health teams^9^, and the School Health Program^10^ (responsible for health promotion and disease prevention actions in Brazil), as well as the publication the Dietary Guidelines for the Brazilian Population, which recommends that ultra-processed foods be avoided, including SB^12^. However, most of these initiatives have been weakened or even stopped after the political and economic crisis experienced by the country since 2015^13,14,19^. Our results confirm our original hypothesis that this change could affect the trend of reduction in SB consumption initially observed. Our five-year update to the previous findings (adding information from 2017 to 2021) reinforced the decreasing trend originally observed for the entire period (2007–2021), but also allowed us to identify that this trend has weakened, without a significant variation in the most recent period (2015–2021).

This change of scenery observed in Brazil since 2015, characterized by reduced investments in social and health policies and increased unemployment and inflation, not only has impacted the reduction in SB consumption but also precludes the adoption of measures to promote the continuity of the decreasing trend, such as the taxation of SB, the imposition of marketing restrictions or even restrictions on the commercialization and consumption of SB in schools and public offices^20-22^. In fact, Brazil is still one of the countries that subsidizes the SB industry with wide tax incentives^23^. However, in an unprecedented move, a law proposal has established a 20% increase in taxation on SB commercialization and imports. The project has been approved in the first instances and is pending in the Federal Senate^15,24^. It is believed that the approval of such a project would be an important step towards reducing the consumption of SB in Brazil, directly impacting the health of the population.

In 2021, 14.0% of the Brazilian adult population consumed SB five days a week, with an average of 287.4 mL/day. A survey of sugar-SB consumption among adults of 187 countries reported that the intake was higher in middle-income countries, such as Brazil, when compared with those with high income or low income^25^. Solid evidence indicates the deleterious effect of SB consumption on health, due to its association with cardiometabolic outcomes, such as metabolic syndrome, weight gain, type 2 diabetes, and dental caries worldwide^2,3,26^. Following the results presented for SB consumption, a recent population health monitoring showed that Brazilian adults experienced the greatest obesity increase in recent times, from 11.8% in 2006 to 22.4% in 2021—an increase of 90%—while diabetes jumped from 5.5% in 2006 to 9.1% in 2021^17^.

Vigitel data are collected with high quality standards, representative of the adult population in the most relevant urban areas in the country, allowing a detailed investigation on trends over a 15-year period. However, some limitations must be considered. The data is based on self-reported information obtained through telephone interviews, being more susceptible to inaccuracies than dietary consumption directly measured. Moreover, the Vigitel questionnaire does not include other types of mixed juices or nectars; however, it was developed to be applied by telephone interview to large population samples^17^. In addition, this method is used in similar health surveys, such as the Behavioral Risk Factor Surveillance System (BRFSS)^27^, or in surveys of several risk factors, like the WHO STEPwise approach (STEPS)^28^. Furthermore, the good validity and reproducibility of the indicators have been previously verified^29,30^. Another limitation includes the sample restriction to individuals with a landline telephone in the capitals of Brazilian states and FD, which is minimized by the weighting factors that allow extrapolating the results for the total population^17^.

The present study identified a decrease trend in the consumption of SB among Brazilian adults in the 15 years studied (2007–2021). However, this reduction was not observed in the most recent period (2015–2021). The adoption of public policies, mainly regulatory, is needed to reduce consumption of SB, maintaining the trend observed throughout the study period, given the higher prevalence of consumption in specific sociodemographic groups.

## References

[1] World Health Organization (2015). Guideline: sugars intake for adults and children.

[2] Malik VS, Hu FB (2022). The role of sugar-sweetened beverages in the global epidemics of obesity and chronic diseases. Nat Rev Endocrinol.

[3] Zhao L, Zhang X, Coday M, Garcia DO, Li X, Mossavar-Rahmani Y (2023). Sugar-sweetened and artificially sweetened beverages and risk of liver cancer and chronic liver disease mortality. JAMA.

[4] World Health Organization (2017). Tackling NCDs: ‘best buys’ and other recommended interventions for the prevention and control of noncommunicable diseases.

[5] von Philipsborn P, Stratil JM, Burns J, Busert LK, Pfadenhauer LM, Polus S (2020). Environmental interventions to reduce the consumption of sugar-sweetened beverages: abridged Cochrane systematic review. Obes Facts.

[6] Chevinsky JR, Lee SH, Blanck HM, Park S (2021). Prevalence of self-reported intake of sugar-sweetened beverages among US adults in 50 states and the District of Columbia, 2010 and 2015. Prev Chronic Dis.

[7] Costa DVP, Lopes MS, Mendonça RD, Malta DC, Freitas PP, Lopes ACS (2021). Diferenças no consumo alimentar nas áreas urbanas e rurais do Brasil: Pesquisa Nacional de Saúde. Ciênc Saúde Coletiva.

[8] Figueiredo N, Maia EG, Silva LES, Granado FS, Claro RM (2018). Trends in sweetened beverages consumption among adults in the Brazilian capitals, 2007–2016. Public Health Nutrition.

[9] Bortolini GA, Oliveira TFV, Silva SA, Santin RC, Medeiros OL, Spaniol AM (2020). Ações de alimentação e nutrição na atenção primária à saúde no Brasil. Rev Panam Salud Publica.

[10] Brasil (2007). Ministério da Saúde. Secretaria de Atenção Primária à Saúde. Programa Saúde na Escola (PSE). [Internet].

[11] Mielke G, Malta DC (2020). Avaliação e futuro do Programa Academia da Saúde. Rev Bras Ativ Fís Saúde.

[12] Brasil. Ministério da Saúde (2014). Guia alimentar para a população brasileira.

[13] Souza LEPF, Barros RD, Barreto ML, Katikireddi SV, Hone TV, Sousa RP (2019). The potential impact of austerity on attainment of the Sustainable Development Goals in Brazil. BMJ Glob Health.

[14] Malta DC, Silva AG, Teixeira RA, Machado IE, Coelho MRS, Hartz ZMA (2019). Avaliação do alcance das metas do plano de enfrentamento das doenças crônicas não transmissíveis no Brasil, 2011-2022. An Inst Hig Med Trop.

[15] Brasil. Presidência da República (2023). Emenda constitucional n^o^ 132, de 20 de dezembro de 2023. Altera o Sistema Tributário Nacional [Internet].

[16] World Health Organization (2022). WHO manual on sugar-sweetened beverage taxation policies to promote healthy diets.

[17] Brasil. Ministério da Saúde (2022). VIGITEL Brasil 2021: vigilância de fatores de risco e proteção para doenças crônicas por inquérito telefônico.

[18] Caldeira TCM, Silva LES, Sousa TM, Soares MM, Claro RM (2023). Temporal trend in the coexistence of risk behaviors for noncommunicable diseases in Brazil: 2009-2019. Prev Chronic Dis.

[19] Silva AG, Teixeira RA, Prates EJS, Malta DC (2021). Monitoramento e projeções das metas de fatores de risco e proteção para o enfrentamento das doenças crônicas não transmissíveis nas capitais brasileiras. Ciênc Saúde Coletiva.

[20] Brasil. Senado Federal (2019). Institui Contribuição de Intervenção no Domínio Econômico incidente sobre a comercialização da produção e da importação de refrigerantes e bebidas açucarados (Cide-Refrigerantes), e dá outras providências [Internet].

[21] Brasil. Ministério da Saúde (2010). Agência Nacional de Vigilância Sanitária. Resolução–RDC n^o^ 24, de 15 de junho de 2010. Dispõe sobre a oferta, propaganda, publicidade, informação e outras práticas correlatas cujo objetivo seja a divulgação e a promoção comercial de alimentos considerados com quantidades elevadas de açúcar, de gordura saturada, de gordura trans, de sódio, e de bebidas com baixo teor nutricional, nos termos desta Resolução, e dá outras providências [Internet].

[22] Brasil. Câmara dos Deputados (2017). Projeto de Lei n^o^ 1755/2007. Dispõe sobre a proibição da venda de refrigerantes em escolas de educação básica [Internet].

[23] Peres J (2017). Toma essa: os bilhões que damos todos os anos à indústria de refrigerantes [Internet]. O Joio e o Trigo.

[24] Mariath AB, Martins APB (2021). Década da Ação em Nutrição e tributação de bebidas açucaradas no Brasil: onde estamos?. Cad Saúde Pública.

[25] Singh GM, Micha R, Khatibzadeh S, Shi P, Lim S, Andrews KG (2015). Global, regional, and national consumption of sugar-sweetened beverages, fruit juices, and milk: a systematic assessment of beverage intake in 187 countries. PloS One.

[26] Valenzuela MJ, Waterhouse B, Aggarwal VR, Bloor K, Doran T (2021). Effect of sugar-sweetened beverages on oral health: a systematic review and meta-analysis. Eur J Public Health.

[27] Centers for Disease Control and Prevention (2020). Behavioral Risk Factor Surveillance System. About BRFSS [Internet].

[28] World Health Organization (2001). The WHO STEP wise approach.

[29] Monteiro CA, Moura EC, Jaime PC, Claro RM (2008). Validade de indicadores do consumo de alimentos e bebidas obtidos por inquérito telefônico. Rev Saúde Pública.

[30] Mendes LL, Campos SF, Malta DC, Bernal RTI, Sá NNB, Velásquez-Meléndez G (2011). Validity and reliability of foods and beverages intake obtained by telephone survey in Belo Horizonte, Brazil. Rev Bras Epidemiol.

